# H_3_^+^ as an ionospheric sounder of Jupiter and giant planets: an observational perspective

**DOI:** 10.1098/rsta.2018.0404

**Published:** 2019-08-05

**Authors:** Pierre Drossart

**Affiliations:** Lesia, Observatoire de Paris-PSL, CNRS, Sorbonne Université, Université de Paris, Paris, France

**Keywords:** planetary atmospheres, infrared spectroscopy, Solar System, exoplanets

## Abstract

Thirty years of observations of ${H_{3}}^{+}$ on Jupiter have addressed many complex questions about the physics of the ionospheres of the giant planets. Spectroscopy, imaging and imaging spectroscopy in the infrared have allowed investigators to retrieve fundamental parameters of the ionosphere, overcoming the inherent limitations and complexities in radiative transfer, and these results are now introduced as model constraints for upper atmospheric structure and dynamics. This paper will focus on the mid-latitude emissions, which are fainter and less well studied than the auroral regions. A new analysis of VLT/ISAAC spectral imaging observations of Jupiter obtained in 2000 at 3.5 µm is presented and discussed in comparison with previous observations to show the spatial distribution of ${H_{3}}^{+}$ emissions compared with other atmospheric structures. Cylindrical maps of Jupiter in three different selected wavelengths show the spatial variations at different altitudes in the atmosphere, from cloud level up to the ionosphere. Evidence for fluctuations in the ${H_{3}}^{+}$ emissions could be due to the presence of stationary or dynamic processes. If the exact origin of these phenomena remains unidentified, several plausible mechanisms are proposed to explain the observed energy deposition and variability: future observation campaigns should deepen the understanding of these complex phenomena, in order to prepare for the future ESA/JUICE mission.

This article is part of a discussion meeting issue ‘Advances in hydrogen molecular ions: H_3_^+^, H_5_^+^ and beyond’.

## 1. Introduction

The history of the spectroscopic discovery of ${H_{3}}^{+}$ on Jupiter, its molecular spectroscopic characteristics and its use as an ionospheric tracer have been reviewed in [1,2]. Only general facts relevant to the present study will be repeated here, in the new perspective of constraining models of upper atmospheric structure for the observations at mid-latitudes of Jupiter and the giant planets. It has been known since the first observations of the 2 µm emissions of ${H_{3}}^{+}$ in the overtone 2*ν*_2_ band [3] that access to ionospheric emissions on Jupiter in the infrared (IR) from ground-based telescopes would give a powerful new tool to constrain auroral emissions, which were limited previously to ultraviolet (UV) observations from space. This detection in the overtone band of ${H_{3}}^{+}$ was rapidly followed by observations in the fundamental band at 3.5 µm [4], and then imaging of the emission in IR filters [5].

In this paper, after a short review in §2 of previous observations and their importance for the understanding of the processes in Jupiter's upper atmosphere, §3 will review the measurements and methods of retrieval of physical atmospheric parameters with their limitations and uncertainties. Then, in §4, observations of mid-latitudes in ${H_{3}}^{+}$ emissions will be discussed. A first analysis in this region of the giant planet Very Large Telescope array (VLT) observations obtained in 2000 will be presented. These observations, which accurately map Jupiter's emission in the L band, will be compared with other observational sets. The modelling approach to interpret the mid-latitude emissions will be discussed in §5, and perspectives for future observations and extension to other planets will be given in the conclusion.

## 2. From first detections to a new sounder of Jupiter

In 30 years of observations in ${H_{3}}^{+}$, spectral and imaging studies have been produced from many large telescopes around the world, e.g. CFHT [3,4], UKIRT [6], IRTF [5], etc., as well as from space instrumentation in Earth orbit telescopes or space missions, e.g. ISO/SWS [7], Galileo/NIMS [8], Cassini/VIMS [9] and JUNO/JIRAM [10]. The corpus of observations is therefore now very large, but the variability of the emissions poses many problems. The temperatures, density and morphology of the ${H_{3}}^{+}$ emissions when observed at a given date are not necessarily applicable for comparison with another set of observations (e.g. UV aurorae versus IR aurorae), as all of these parameters have been observed to be time variable.

Coordinated campaigns, in addition to dedicated observations focused on ${H_{3}}^{+}$, have to be prepared to compare UV emissions in H and H_2_ with ${H_{3}}^{+}$ in the IR, X-ray emissions or radio emissions for Jupiter, even if ${H_{3}}^{+}$ dedicated campaigns remain valuable. It is therefore understandable that many mysteries remain about the production and evolution of the emissions. In this paper, the focus is on the mid-latitude (non-auroral) ${H_{3}}^{+}$ emissions, which are usually 20 times fainter than typical auroral emissions and are, therefore, more difficult to study.

One of the main objectives of the spectral or spectral imaging observations of ${H_{3}}^{+}$ is to constrain and interpret the planetary phenomena related to the emissions. The accessible measurable parameters are:

— the temperature of the ionosphere (from rotational and vibrational distributions or line width measurement);
— the column density of ${H_{3}}^{+}$;
— the wind velocity of the ionosphere from Doppler shift measurement;
— the spatial and temporal variability, to correlate with other measurements;
— the altitude of the emissions from direct limb observations in imaging at high spatial resolution;
— the morphology of the spatial distribution of ${H_{3}}^{+}$ emissions to constrain the magnetic field model of Jupiter [11];
— the multi-wavelength correlation: X-ray, UV, IR, radio to correlate with magnetospheric, solar wind or internal dynamic processes;
— the energy balance, from the total estimated IR emission from ${H_{3}}^{+}$ to be compared with energy inputs from external or internal sources.

This corpus of parameters, when entered in planetary models such as the Thermal Global Circulation Models [12,13] or auroral precipitation models [14,15], constrains the atmospheric physics and allows us to refine our global understanding of the dynamics of the ionosphere. In this domain, very different processes are at work between auroral processes and mid-latitude processes. This paper will address mostly the latter, auroral processes being addressed in other reviews [16,17].

## 3. Parameter retrieval: limitations and complexities

The interpretation of the observations starts with a comparison with a radiative transfer model. A great simplification in the model of ${H_{3}}^{+}$ emissions comes from the low optical thickness of the emission, which comes from the ionosphere well above any other Jupiter emissions. This emission is therefore an additive component to Jupiter's spectrum, and, contrary to the common process of thermal emission retrieval in planetary atmospheres, the molecular emissions of ${H_{3}}^{+}$ can easily be extracted independently of other processes. It can be asked if the ${H_{3}}^{+}$ emission downward to the Jupiter clouds could not be reflected by the clouds and then contribute to the global emission. This process is nevertheless hindered by the large optical depth of the atmosphere between the ionosphere and the upper cloud layers of Jupiter, which essentially reabsorb the emissions. This is particularly true for L band observations at 3.5 µm, where methane absorptions are huge to the point that they almost cancel any cloud reflection. The underlying hypothesis of the retrieval is described below.

### (a) Optical thickness

The question of the optical thickness of the emissions is relatively simple to answer at first order: as many authors have shown, the column density retrieved in ${H_{3}}^{+}$ is of the order of 10^12^ cm^2^ in auroral regions [18], and much less outside. The radiative transfer is therefore simplified, the emitted flux for a given molecular transition being proportional to the column density N* of ${H_{3}}^{+}$ in the upper level, multiplied by the spontaneous emission coefficient (Einstein coefficient) of the transition. Stimulated emission or absorption can be neglected because of the low optical thickness.

In the case of local thermal equilibrium (LTE) and uniform temperatures (these hypotheses are discussed below), N* is simply given by: N* = *N* exp(−*E_j_*/*kT*), where *N* is the total column density of ${H_{3}}^{+}$, *E_j_* is the energy of the upper level, *T* is the temperature and *k* is the Boltzmann constant. The two parameters *N* and *T* can therefore be retrieved if enough information is present in the spectrum. In principle, two lines should be enough for this retrieval, on the condition that they have different energies in the upper level and a high enough signal to noise (S/N) ratio. In less than ideal conditions (proximity of *E_ij_*, low S/N), the uncertainties in the retrieval can be large for independent determination of temperature and column density. A globally less constrained parameter, with better S/N determination, is the total emission in a line of ${H_{3}}^{+}$ [19]. When searching for spatial variations, the accuracy of the independent retrieval of *T* and *N* can be affected and careful analysis is required to ensure that spatial maps are really independent.

### (b) Vertical homogeneity

The temperature gradient in the ionosphere is usually very large, as models and direct Galileo observations [20] have shown: it is therefore oversimplifying to assume a constant temperature for the emission. Vertical profiles of ${H_{3}}^{+}$ can only be obtained from ionospheric models, and can be parametrized with a limited number of constants (ideally only one): the modelling in such a case is similar to the previous modelling with two parameters describing the temperature at a given level and column density. Since the predicted ionospheric profiles peak at a given altitude, the simple model is nevertheless acceptable at a first level of approximation, because contributions from levels above the peak are less important because of an exponential decrease in the density, and from levels below the peak because of a lower temperature also inducing an exponentially decreasing intensity in the emission owing to the Boltzmann factor.

### (c) Non-LTE processes

The presence of non-LTE processes is more complex as the retrieval described above assumes equilibrium temperatures between ${H_{3}}^{+}$ emissions to be correct. Non-LTE effects appear when the collision frequency becomes comparable to the radiative de-excitation frequency. As the density decreases in the upper atmosphere, these effects appear therefore necessarily at some levels in the atmosphere. Different approaches have tested the hypothesis, as follows.

— Rotational/vibrational temperatures: the two temperatures can be measured independently if two vibrational bands are observed simultaneously. As the vibrational levels are well separated, it is not easy to obtain simultaneously both *ν*_2_ and 2*ν*_2_ bands for example. Fortunately, hot bands of ${H_{3}}^{+}$ are detectable in the L band and in the same domain that *ν*_2_ and 2*ν*_2_−ν_2_ bands are observable, permitting the vibrational and rotational temperature levels to be tested. An accurate discussion of these phenomena is given in [21,22]. The results show significant differences [23] and non-negligible effects in the vibration bands of ${H_{3}}^{+}$.
— Kinetic temperature: this can be directly measured through the Doppler width of the ${H_{3}}^{+}$ line. This was done initially by Drossart *et al.* [24], and confirmed more recently with additional details by Giles *et al.* [25]. To retrieve the Doppler line width, the combination of the instrumental line shape and rotation broadening must be disentangled. For observations at spectral resolutions larger than 10^5^ and a kinetic temperature of 1000 K, the thermal, instrumental and rotation broadenings are, respectively, 0.037, 0.029 and 0.014 cm^−1^. A proper convolution model of the three effects gives access to the kinetic temperature [26]. Kinetic temperatures are found to be higher than the usual rotational temperatures and a systematic effect seems to be present, and departure from the LTE is suspected to be the cause of this effect.

Again, even if first-order calculation is not disqualified by the results, it is certain that accurate modelling needs to take these different effects into account to properly retrieve the atmospheric parameters [23]. Once the atmospheric parameters are extracted, they can be used as inputs for interpretation models of the physics of the atmosphere. To constrain such models, the most useful methods are obtained from two-dimensional imaging observations where areas of emission are observed: this has been extensively used in auroral observations [23,26]. Auroral models are specific and their relation to the magnetosphere is very different from their relation to the mid-latitudes. Auroral emissions are discussed in Moore *et al.* [17] and Dinelli *et al*. [16].

## 4. New insight into the modelling of Jupiter's atmosphere

Mid-latitude ${H_{3}}^{+}$ emissions are more difficult to observe because of a factor as large as 100 between the bright auroral emission and the faint average mid-latitude emissions. A large sensitivity is therefore needed for such observations and only a few observations have been available to date [27–29]; also discussed in Moore *et al.* [17] and Ray *et al.* [30].

**Figure 1.**
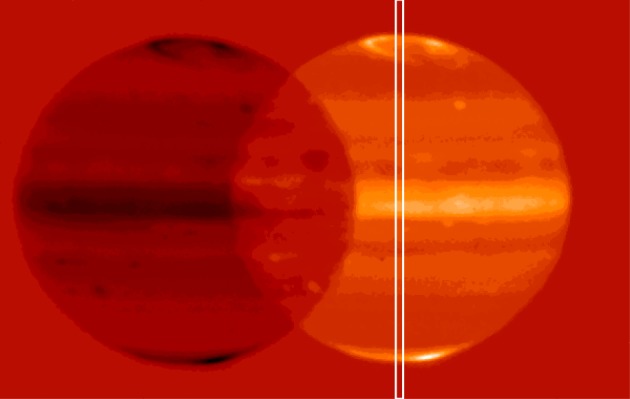
Configuration of observations (VLT/ISAAC Jupiter image with the L filter) [32]. The 1 arcsec slit position is indicated, aligned with the central meridian of Jupiter (1024 × 1024 detector with 0.146 arcsec/pixel). Chopping/nodding is applied for proper sky subtraction, in the transverse direction (the ‘negative’ image of Jupiter shows the spatial extension of the chopping, which does not affect the slit spectra).

Data reductions for mid-latitude observations are presented here for the first time, completing the observations listed above. Jupiter observations were obtained using the ISAAC instrument of VLT [31] on 13 and 14 December 2000 (figure 1). The IR detector has a pixel size on the sky of 0.146 arcsec/pixel and is used for spectral imaging in the 3.3–3.5 µm range at a spectral resolution of approximately 2000. The chopping/nodding mode is applied with 30 arcsec amplitude in the direction perpendicular to the slit. The time between two spectral images is about 10 min, with co-addition at a fixed position in the interval, and the repetition of the observations with a fixed slit along a Jupiter's meridian gives a spatial longitudinal map due to the Jovian rotation. Figure 1 presents the observation strategy with ISAAC: the slit was north/south aligned along the central meridian of Jupiter. Observations on the two nights cover a contiguous longitude range from 230° to 360° and from 0° to 150° in System III longitude. It is unfortunate that the Great Red Spot was outside the covered range, missing the detection of anomalous emissions in ${H_{3}}^{+}$ reported by O'Donoghue *et al.* [29].

**Figure 2.**
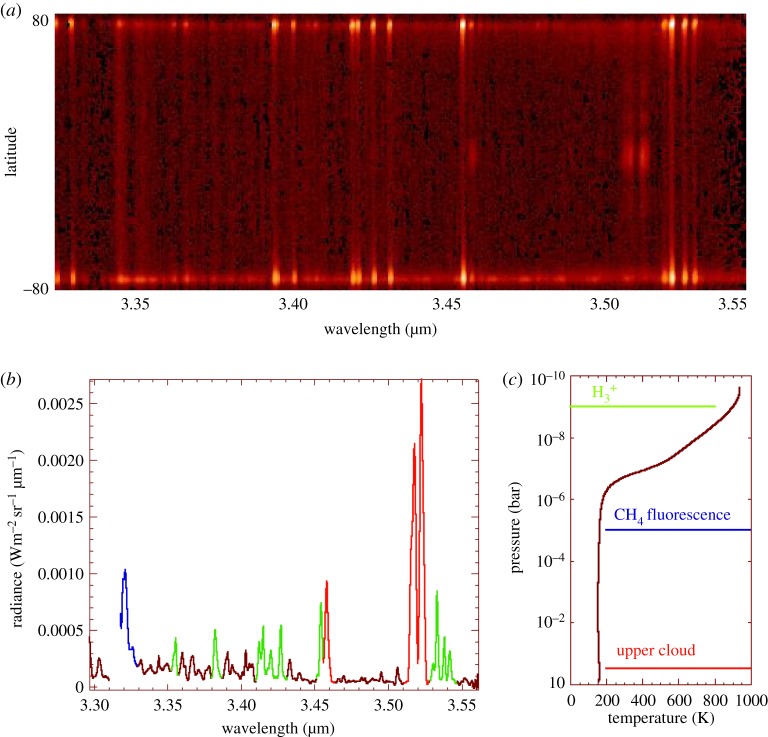
(*a*) Two-dimensional spectral image from ISAAC in the 1 arcsec slit: the 3.3–3.5 µm range (horizontal) is covered at a resolution of 2000, with an integration time of approximately 10 min/spectrum (5 min on target)—star calibration is obtained on BS1380/HD28099. The spatial dimension (vertical) covers the Jovian central meridian from north (top) to south (bottom). (*b*) Mid-latitude spectrum showing the different parts of the emissions: the summation of the spectra at mid-latitudes (between 40 N and 40 S) is shown on the spectrum. Three different spectral features are observed in the atmosphere of Jupiter: ${H_{3}}^{+}$ lines (green), CH_4_ lines (blue), corresponding to the fluorescence of methane [33], and cloud top reflection (red) in narrow spectral windows [34]—the *y*-axis is in radiance units. (*c*) Thermal profile of Jupiter from [20]: the temperature/pressure variation ranges from 1 bar to 0.1 nbar; the altitude of the peak emission or reflection observed in the spectrum of (*b*) is plotted.

Figure 2 shows how spectral images are retrieved from the original ISAAC spectral image (figure 2*a*): an average spectrum is co-added over all mid-latitudes (figure 2*b*). The spectrum shows three characteristic spectral features, from very different atmospheric origins mapped in figure 2*c*.

— Cloud reflections are observed in sharp windows in the otherwise very broad methane absorption by the *ν*_3_ band [34]—the solar reflection on the cloud, located at approximately 300 mbar, is typical of the methane band images of Jupiter, the cloud top being higher in the equatorial and polar regions.
— Methane emissions from *ν*_3_ and hot bands produced by the fluorescence of methane in the atmosphere of Jupiter [33]: the typical altitude of these emissions is estimated in the 0.1–1 mbar range.
— ${H_{3}}^{+}$ emissions from ionospheric altitudes.

**Figure 3.**
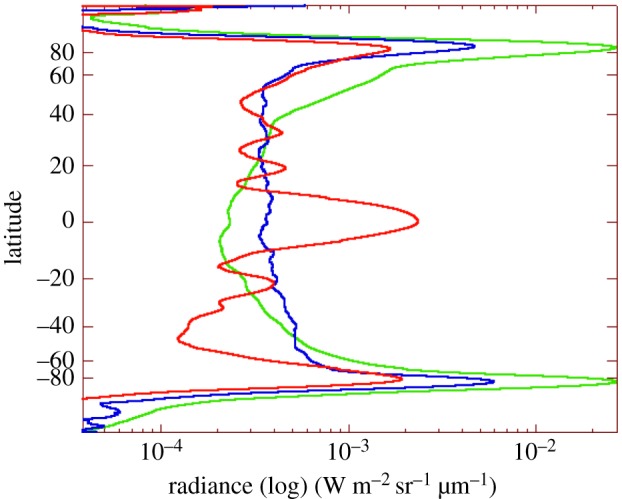
North/south radiance profiles in the three components of figure 2*b*; a global average in longitude has been made to produce these complete profiles. The large contrast between ${H_{3}}^{+}$ auroral and mid-latitude emissions (green) is apparent in the radiance log scale plotted in the abscissae. The fluorescent emission (blue) is flat at mid-latitude, as expected from the model of a volume emission controlled by solar absorption in an optically thin regime—the increase in the polar region is due to contamination by faint ${H_{3}}^{+}$ lines. The cloud reflection at 3.52 µm (red) shows the typical structure of the upper atmospheric haze in methane band images (equatorial and polar hazes).

The spatial variation of the different components is given in figure 3 for the three observed layers. The zonally averaged emission is plotted in radiance (log) units in the abscissae, versus latitude, which exhibit very different behaviour for each component.

**Figure 4.**
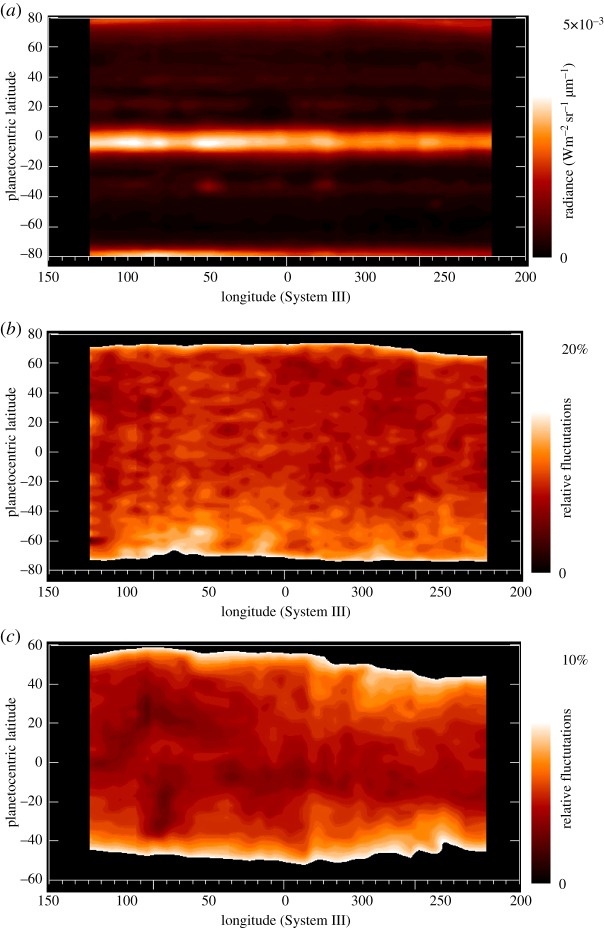
Reconstructed cylindrical maps (for System III longitudes between 230°–360° and 0°–150°) for the three components plotted in figure 3. (*a*) Cloud structure (radiance units). The cloud top observed on Jupiter with the L filter is very homogeneous in longitude; the equatorial band and polar haze are the most prominent structures in latitude. (*b*) Fluorescence map (radiance fluctuation in %). Fluctuations are calculated by subtracting the average radiance map. The homogeneity of the map with fluctuations lower than 20% gives a limit in the eddy diffusion coefficient lower than 30% according to the model of fluorescence [33]. (*c*) ${H_{3}}^{+}$ map (radiance fluctuations in %). As in (*b*), a subtraction of the average emission is performed. Owing to the large contrast (greater than 100) between the auroral and mid-latitude regions shown in figure 3*b*, the latitude range has been limited to ±60° to focus on the mid-latitude variations. Fluctuations are also observed at a 10% amplitude, which corresponds to variations in temperatures lower than 30 K in a simple retrieval model.

— The solar reflection is correlated with the cloud altitude, and has the usual aspect of Jupiter in methane band filters. A discussion of the observations at this wavelength is developed in [34], and is relevant to stratospheric studies, well below the upper atmosphere emissions discussed here.
— The fluorescence of methane is fairly uniform and shows little variation from pole to pole (a polar enhancement is suspected to be due to contamination by faint ${H_{3}}^{+}$ lines at the same wavelengths).
— The ${H_{3}}^{+}$ band has a limb brightening structure, typical of optically thin emissions, and exhibits strong auroral enhancement at high latitudes.
— Figure 4 shows the reconstructed cylindrical maps (for longitudes ranging from 230° to 360° and from 0° to 150° in System III longitude) for the three components.
— Figure 4*a*: cloud structure. The cloud top observed on Jupiter in the L filter is very homogeneous in longitude; the equatorial band and polar haze are the most prominent structures in latitude.
— Figure 4*b*: fluorescence maps. Fluctuations are above the noise level, and may be due to local turbulence in the atmosphere. The model of CH_4_ fluorescent emission [33] is sensitive to the eddy diffusion coefficient, which limits the vertical extension of methane below the homopause level. The homogeneity of the map with fluctuations lower than 20% implies that the variation of the eddy diffusion coefficient is limited to being lower than 30% according to the model.
— Figure 4*c*: an ${H_{3}}^{+}$ map of fluctuations is presented after subtraction of the average emission as in figure 4*b*. Limitation to the mid-latitudes below 60° has been chosen as the factor of 10 in flux intensity in north and south aurorae (not discussed here) compared with equatorial intensity, making it difficult to present both emissions together on a linear scale. Fluctuations are observed at a 10% amplitude, which corresponds to variations in temperatures lower than 30 K in a simple retrieval model. Compared with the most recent analysis [35], the present analysis is unfortunately not complete enough to map the emissions against magnetospheric variation and gives only a global trend on the existence of variation. This map nevertheless shows some interesting aspects when compared with previous observations by Stallard *et al*. [28]: the dark patches observed at 100° longitude show some similarity between the two sets of observations. These similarities are expected if related to the magnetic field structure, as detailed in [28]. North/south asymmetry may be present, but this is close to the S/N level, and comparison with future and more sensitive observations would be needed to confirm this point.

The variations of ${H_{3}}^{+}$ spatial distribution can have two different origins, as follows.

— Static structure, related to magnetospheric variations or other inhomogeneities in particle precipitations.
— Fluctuations (temporal and spatial) related to wave propagation in the atmosphere affecting ${H_{3}}^{+}$ emissions.

Since the observations shown in figure 4 cannot be repeated, it is not possible to ensure that the fluctuations are due to temporal variations (waves) or spatial variations (structural). A discussion of the two models is given in §5.

## 5. Interpretation model

Two categories of models can explain the energy deposition in the high atmosphere of Jupiter leading to high exospheric temperatures: external forcing by precipitation of particles, X-ray and UV solar flux, or internal forcing by the dissipation of internal waves (gravity waves (GWs), acoustic waves or planetary waves). Both mechanisms have been demonstrated to be present in the mid-latitude atmosphere of Jupiter, but their relative importance is still largely debated today.

### (a) External forcing

At low latitudes, ${H_{3}}^{+}$ is expected to be produced predominantly from solar extreme UV (EUV) ionization of H_2_, but particle precipitation could also be present. X-ray emissions have been detected from the ROSAT satellite and an interpretation given by Waite *et al*. [36] relates this emission to ions, in particular sulfur and oxygen ions from the inner magnetosphere of Jupiter. An alternative explanation, supported by observations from the Chandra satellite, is from the scattering of solar X-rays by the atmosphere of Jupiter [37]. Both mechanisms have been shown to be present, but the relative importance of them is debated. A global model of magnetosphere–ionosphere coupling [38] shows that thermospheric temperatures can vary by 20–175 K owing to magnetospheric compression or expansion due to solar wind interaction with the Jovian magnetosphere.

### (b) Internal forcing

GWs were directly observed and characterized *in situ* by the Galileo probe in 1996 [39]: the accelerometer observations were interpreted by the presence of two waves, most probably interpreted as GWs from frequency/wavelength analysis. Observations from radio-occultation by various missions (Voyager, Galileo), e.g. [40], give a vertical distribution of the electronic density exhibiting large fluctuations interpreted as being due to GWs. A third set of observations is from stellar occultation measurements [41], where the atmospheric density is sounded vertically at high spatial resolution: density fluctuations again are identified as being consistent with GWs. The measurement of the vertical temperature gradient shows a lower limit corresponding to the Brunt–Vaisala frequency, interpreted as the threshold of convective dissipation of the wave energy, similar to Earth observations [42].

A model linking GW propagation to ${H_{3}}^{+}$ density/temperature variations has been produced by Barrow & Matcheva [43] and Barrow *et al*. [44]. Taking the GW parameters observed by Galileo as a realistic starting point for amplitude and wavelength propagation parameters, a complete model of ${H_{3}}^{+}$ density and temperature fluctuation is built in the ionosphere: the prediction of observable effects above a few per cent gives an interesting indication that GWs are indeed detectable from ${H_{3}}^{+}$ accurate observations.

Observations have been made of ${H_{3}}^{+}$ emissions located above the Great Red Spot of Jupiter [29], and Ray *et al.* [30] give support to the possibility of internal wave forcing, contributing to a heating in the ionosphere observable in ${H_{3}}^{+}$. The characterization of the wave responsible for the emissions is nevertheless difficult: ideally, a temporal/spatial survey should be obtained to measure at least the wavelength and period and to compare them with observations. Such observations are difficult and not presently available. Only a partial deduction can therefore be made today, on the observability of the effects. On the other hand, links between the radiation belt and the thermosphere have been demonstrated [45], providing clues that external effects are present in the generation of ${H_{3}}^{+}$ variations. The model and joint observations of the Jovian synchrotron radiation and ${H_{3}}^{+}$ IR emissions rely on the variation in solar UV/EUV heating, the Jovian synchrotron radiation and thermospheric temperature variations observed through ${H_{3}}^{+}$.

The corpus of current observations clearly demonstrates the presence of both sources of energy (internal and external) to generate ${H_{3}}^{+}$ emission variation: only long-term observations of temporal fluctuations will in the future allow these observations to be extended to a full and comprehensive dataset for interpretation.

## 6. Conclusion: perspectives and future observations

The interest in mid-latitude observations of ${H_{3}}^{+}$ emissions in Jupiter has been shown to be related to fundamental questions in planetary physics. These observations are complementary to auroral observations, which are now regularly observed by the JUNO mission [46] and have been reported by Dinelli *et al.* [16]. JUNO will unfortunately not be as efficient in the study of mid-latitude fainter ${H_{3}}^{+}$ emissions, and the orbital geometry of the mission is not ideal for such studies. The ESA JUICE mission will provide a space observatory of high interest to address this question. Nevertheless, ground-based observations are still highly valuable, at the condition of observing on a long time scale to retrieve not only spatial variations as already observed in previous observations, but also temporal variations. It therefore needs consequent telescope time—a support to the JUICE mission may be a good frame for such observations in the future.

Finally, Jupiter has been described in this article as the most studied and most accessible giant planet, but comparison with Saturn and Uranus is of course of high interest for a complete understanding of the physical processes in ${H_{3}}^{+}$. A long-term goal is also the detection of ${H_{3}}^{+}$ in exoplanets; today, only an upper limit on ${H_{3}}^{+}$ emissions has been obtained in exoplanets to date [47], but future observations in transit spectroscopy, or direct detection, could lead to their detection. The ARIEL space observatory [48] will be a main tool in this search by the mid-2020s.

## Data accessibility

New data are presented from ESO/VLT ISAAC 66.C-0070(A) observations of Jupiter on 13 and 14 December 2000. The program description and data are publicly available on the ESO website: http://archive.eso.org/eso/eso_archive_main.html. A query can be made under program 66.C-0070(A) in order to access the data. A standard procedure of data reduction is applied through the owned program (flat fielding, sky subtraction, stellar flux calibration on star BS1380/HD28099 and geometry calculation).
